# Thrombin Inhibition Reduces the Expression of Brain Inflammation Markers upon Systemic LPS Treatment

**DOI:** 10.1155/2018/7692182

**Published:** 2018-06-19

**Authors:** Efrat Shavit Stein, Marina Ben Shimon, Avital Artan Furman, Valery Golderman, Joab Chapman, Nicola Maggio

**Affiliations:** ^1^Department of Neurology, The Chaim Sheba Medical Center at Tel HaShomer, 52621 Ramat Gan, Israel; ^2^Department of Neurology and Neurosurgery, Sackler Faculty of Medicine, Tel Aviv University, 6997801 Tel Aviv, Israel; ^3^Talpiot Medical Leadership Program, The Chaim Sheba Medical Center at Tel HaShomer, 52621 Ramat Gan, Israel; ^4^Sagol School of Neuroscience, Tel Aviv University, 6997801 Tel Aviv, Israel

## Abstract

Systemic inflammation and brain pathologies are known to be linked. In the periphery, the inflammation and coagulation systems are simultaneously activated upon diseases and infections. Whether this well-established interrelation also counts for neuroinflammation and coagulation factor expression in the brain is still an open question. Our aim was to study whether the interrelationship between coagulation and inflammation factors may occur in the brain in the setting of systemic inflammation. The results indicate that systemic injections of lipopolysaccharide (LPS) upregulate the expression of both inflammatory and coagulation factors in the brain. The activity of the central coagulation factor thrombin was tested by a fluorescent method and found to be significantly elevated in the hippocampus following systemic LPS injection (0.5 ± 0.15 mU/mg versus 0.2 ± 0.03 mU/mg in the control). A panel of coagulation factors and effectors (such as thrombin, FX, PAR1, EPCR, and PC) was tested in the hippocampus, isolated microglia, and N9 microglia cell by Western blot and real-time PCR and found to be modulated by LPS. One central finding is a significant increase in FX expression level following LPS induction both in vivo in the hippocampus and in vitro in N9 microglia cell line (5.5 ± 0.6- and 2.3 ± 0.1-fold of increase, resp.). Surprisingly, inhibition of thrombin activity (by a specific inhibitor NAPAP) immediately after LPS injection results in a reduction of both the inflammatory (TNF*α*, CXL9, and CCL1; *p* < 0.006) and coagulation responses (FX and PAR1; *p* < 0.004) in the brain. We believe that these results may have a profound clinical impact as they might indicate that reducing coagulation activity in the setting of neurological diseases involving neuroinflammation may improve disease outcome and survival.

## 1. Introduction

A growing body of evidence links systemic inflammation and disease pathogenesis in the brain [1–3]. Upon stimulation by endogenous (e.g., injury, stroke, and autoimmune processes) or exogenous challenges (e.g., pathogens or severe psychological stressors), the immune system upregulates the expression of several cytokines in the brain [4–10]. This results in microglia alteration and ultimately disruption of the delicate neuroglial interactive balance causing alteration of cognition and behavior [11, 12]. Brain inflammation has as well been linked to neuronal damage and neurodegeneration [2, 13, 14].

In the periphery, systemic inflammation has been shown to alter the expression of blood coagulation factors [15]. Indeed, in either autoimmune and/or severe infections (i.e., sepsis), inflammation and coagulation systems are simultaneously activated [16]. A crosstalk among them amplifies and maintains their activation with profound local and systemic implications [17]. Thrombin, the main coagulation factor, has pronounced proinflammatory effects [18]. Acting via specific cell membrane receptors, the protease-activated receptors (PARs), which are abundantly expressed in all arterial vessel wall constituents, thrombin has the potential to exert proatherogenic actions, such as leukocyte migration, cellular proliferation, regulation of vascular permeability and tone, platelet activation, and edema formation [19–23].

Thrombin and its inactive precursor prothrombin have been also detected in the brain [24, 25]. Although the precise cellular source of thrombin in the brain and the molecular mechanisms responsible for its formation and release warrant further investigation, experimental evidence has been provided that neural prothrombin expression and thrombin activity are highly regulated under physiological and pathological conditions [24, 26].

While several evidences point out at an interrelation between systemic inflammation and coagulation, to date, there are no proofs on whether neuroinflammation could interact with the expression of coagulation factors in the brain.

In this manuscript, we investigated whether an interrelationship between coagulation and inflammation factors may occur in the brain in the setting of systemic inflammation [2, 13, 14, 27]. Our data show that systemic injections of lipopolysaccharide (LPS, a component of the bacterial wall) upregulate the expression of both inflammatory and coagulation factors in the brain. Surprisingly, inhibition of thrombin activity prior to LPS injection results in a reduction of both the inflammatory and coagulation responses in the brain. We believe that these results may have a profound clinical impact as they might indicate that reducing coagulation activity in the setting of neurological diseases involving neuroinflammation may improve disease outcome and survival.

## 2. Materials and Methods

### 2.1. Experimental Setting

The experiments were approved by the Institutional Animal Care and Use Committee of the Sheba Medical Center which obeys to the national- and NIH-approved rules (1000/15). The minimal number of animals was used, and all efforts were made to minimize suffering. The study was carried out in 8-week-old male C57BL/6 mice, purchased from Envigo Laboratories, Israel. Mice were injected IP (intraperitoneal) with LPS (*Escherichia coli* 0111:B4, Sigma L4130, 1 mg/kg, diluted in saline) only, LPS and NAPAP (Sigma Pefabloc 76308, 0.75 mg/kg), NAPAP or 150 *μ*l saline only (*n* = 6 for each group). 24 hrs following the injection, mice were anesthetized with pentobarbital (0.8 mg/kg) and the brains were removed for hippocampus dissection.

### 2.2. Cell Cultures

The microglial cell model murine microglial cell line N9 was used [28]. Cells were a generous gift from Professor Dan Frenkel of Tel Aviv University. Cells were grown in RPMI 1640, supplemented with 10% fetal bovine serum, 1% glutamine, and 1% Pen.-Strep. Cells were kept at 37°C with 5% CO_2_ and 95% relative humidity. For experiments, N9 cells were seeded into 6-well plates at a density of 10^4^ cells per well. The medium was supplemented with 0.1 *μ*g/ml LPS for 24 hrs, then the cells were harvested for protein and mRNA analysis (*n* = 3).

### 2.3. Quantitative PCR

Prior to the harvest, the animals were anesthetized with pentobarbital. The brains were removed and the hippocampi were dissected. The RNA tissue was extracted using the TRIzol (Thermo Fisher 15596026) solubilization method followed by phase separation with chloroform. Samples were placed in 1 ml TRIzol and homogenized with bullet blender homogenizer (Next Advance) at a maximum speed for 1 minute. RNA phase cleaning was performed using Bio-Rad Aurum 732-6820 (Bio-Rad Laboratories, Hercules, CA, USA). N9 cell mRNA was extracted by lysis buffer addition to the cells accordingly the Bio-Rad Aurum 732-6820 kit. Two micrograms of total RNA was used for reverse transcription using high-capacity cDNA reverse transcription kit (Applied Biosystems). Quantitative real-time polymerase chain reaction was performed on the StepOne™ Real-Time PCR System (Applied Biosystems, Rhenium, Israel) using Fast SYBR Green Master (ROX) (Applied Biosystems). Hypoxanthine guanine phosphoribosyltransferase (HPRT) served as a reference gene in this analysis (primer list). A standard amplification program was used (1 cycle of 95°C for 20 seconds (s) and 40 cycles of 95°C for 3 s and 60°C for 30 s. The primers used in this analysis are listed in Table 1. The results were normalized to reference gene expression within the same cDNA sample and calculated using the ΔCt method with results reported as fold changes relative to control brains of sham animals and reported as mean ± SE.

### 2.4. Western Blot

N9 cell line samples (*n* = 3) were lysed in RIPA buffer (containing in mM: 50 TRIS HCl pH 8, 150 NaCl, 1% NP-40, 0.5% sodium deoxycholate, and 0.1% SDS) and a protease inhibitor cocktail (Merck Millipore 539134). The homogenates were centrifuged (13,000*g* × 5 min) at 4°C. The supernatants were collected, and protein concentration was determined through a bicinchoninic acid (BCA) assay. 20 *μ*g from each sample was separated by SDS-polyacrylamide gel electrophoresis. The proteins were transferred onto nitrocellulose membranes. Membranes were incubated with rabbit anti-FX (1 : 1000, BS-77622, Bioss), thrombin (1 : 400, BS-19142, Bioss), PAR1 (1 : 500, BS-0828R, Bioss), EPCR (1 : 500, NBP2-21578 Novous Biologicals), protein C (1 : 400, 251142 Abbiotec), and TNF*α* (1 : 500, gtx-110520) over night at 4°C and washed with tris-buffered saline and 0.1% Tween 20 (TBST). Membranes were then incubated at room temperature with horseradish peroxidase-conjugated goat anti-rabbit antibody (1 : 10,000, Jackson Immunoresearch Laboratories). Protein bands were detected by a peroxidase-based ECL method. Upon detection, the membranes were stripped and reincubated with a mouse anti-HSC70 antibody (1 : 10,000, sc-7298) and redetected by ECL. Analysis of the protein band density was performed with ImageJ software.

### 2.5. Thrombin Activity

Thrombin enzymatic activity was measured using a fluorometric assay based on the cleavage rate of the synthetic substrate Boc-Asp(OBzl)-ProArg-AMC (I-1560; Bachem, Bubendorf, Switzerland) and defined by the linear slope of the fluorescence intensity versus time, as previously described [29, 30]. 24 hrs following LPS injection, mice (*n* = 6) were anesthetized with pentobarbital and brains were removed for hippocampus dissection. The dorsal part of the hippocampus was collected for thrombin-like activity assay. The hippocampal tissue was then placed into 96-well black microplate (Nunc, Roskilde, Denmark) containing the substrate buffer. Measurements were carried out using a microplate reader (Tecan; Infinite 200; Switzerland) with excitation and emission filters of 360 ± 35 and 460 ± 35 nm, respectively. Reported values are normalized to protein concentration of each sample (±SEM).

### 2.6. Microglia Cell Separation

Microglia cells were isolated from hippocampus homogenates. Mice were anesthetized with ketamine/xylazine solution (100 mg/kg and 18.6 mg/kg, resp.) and perfused with cold PBS. The brains were removed and the hippocampi from two mice were pooled. The pooled samples (*n* = 3–8) per tested target gene were triturated using mechanical homogenization by syringe, in Hank's Balanced Salt Solution (HBSS), pH 7.4. Resulting homogenates were passed through a 70 *μ*m nylon cell strainer and centrifuged at 975*g*, 4°C for 5 min. Supernatants were removed and cell pellets were resuspended in 40% isotonic Percoll (Sigma, p1644) at room temperature. The gradient was centrifuged for 15 min at 975*g*, and the supernatant was discarded. Cells were washed in staining buffer (at 375*g* for 6 min) and then stained with FITC anti-mouse CD11b (BioLegend, 101205), APC anti-mouse CD45 (BioLegend, 103111), and Pacific blue anti-mouse Ly-6G (BioLegend, 127611). The number of viable cells was determined using a hemocytometer and 0.1% trypan blue staining. Positive microglia cells were collected according to the gating definition: Ly-6G^−^/CD11b^+^/CD45^low^. The positive cells were collected in RNA lysis buffer and RNA was purified immediately (Qiagen RNeasy Plus Micro Kit 74034).

### 2.7. Statistical Analysis

Statistical analysis was performed using the GraphPad Prism 7 software. The statistical comparisons between groups were performed using a Student *t*-test and ANOVA followed by Tukey's multiple comparison test. *p* values ≤ 0.05 were considered significant between means. All results present as mean ± SE of the mean.

## 3. Results

### 3.1. Systemic Injection of LPS Activates the Inflammation and Coagulation Response in Microglia

**Figure 1:**
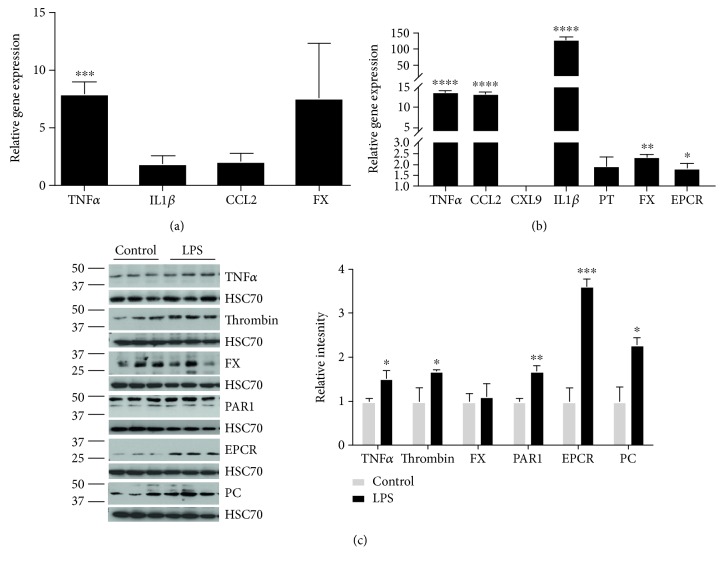
LPS induces inflammation in microglia cells *in vivo* and *in vitro*. Gene expression in mouse hippocampal isolated microglia cells 24 hrs following systemic LPS injection (a). Inflammation and coagulation gene expression in N9 microglial cell line, 24 hrs following LPS treatment (b). Protein expression of coagulation and inflammation factors in N9 microglial cell line. Representative blots are presented on the left panel; each protein of interest normalized to HSC70 protein. The graph represents relative intensities that reported as fold change relative to control samples (c). Results are presented as mean ± SEM. ^∗^*p* ≤ 0.05, ^∗∗^*p* ≤ 0.01, ^∗∗∗^*p* ≤ 0.001, and ^∗∗∗∗^*p* ≤ 0.0001.

Systemic injections of LPS activate the inflammatory response in the periphery and in the brain [31]. Microglia, the brain resident macrophage cells, are the first and main form of active immune defense in the central nervous system. Upon 24 hours from i.p. LPS injections, TNF*α* gene expression level in microglia increased significantly (7.9 ± 0.4-fold, *p* = 0.0006, *n* = 4) as well as those of the cytokine IL1*β* (1.8 ± 0.3-fold, *n* = 8, Figure 1(a)). Chemockine (C-C motif) ligand 2 (CCL2), a gene encoding for a factor recruiting inflammatory cells during the inflammatory response [32], was upregulated (2.1 ± 0.6-fold, *n* = 4) in microglia cells from LPS-treated animals (Figure 1(a)). Interestingly, LPS was able to activate the expression of factor X, a coagulation factor, which increased by 7.5-fold (*n* = 4) in microglia of LPS-treated animals compared to control (Figure 1(a)).

Treating microglia cell lines (N9) with LPS resulted in a similar upregulation of inflammation-related gene expression (Figure 1(b)). In this setting, the expression levels of TNF*α*, IL1*β*, and CCL2*β* were increased (13.8 ± 0.47-, 129 ± 8.5-, and 13.6 ± 0.33-fold, resp., *p* < 0.0001) (Figure 1(b)). TNF*α* protein levels likely raised significantly compared to control (1.5 ± 0.187, *p* = 0.03) following LPS treatment (Figure 1(c)). Interestingly, the coagulation factors were equally affected following LPS treatment in microglia cells (Figures 1(a)–1(c)). The expression of genes for factor X, prothrombin, and EPCR reached levels of 2.34 ± 0.2, 1.9 ± 0.4, and 1.8 ± 0.2 (*p* ≤ 0.01) compared to their corresponding controls, respectively (Figure 1(b)). Coagulation factor protein levels as well increased following LPS treatment of microglia cell lines. Specifically, thrombin, PAR1, EPCR, and PC reached the highest levels of expressions compared to their respective controls (1.6 ± 0.03, 1.6 ± 0.1, 3.6 ± 0.1, and 2.2 ± 0.16, *p* ≤ 0.04) while factor X was lightly upregulated with its value not reaching statistical significance (Figure 1(c)).

All in all, these data indicate that upon LPS challenge, inflammation and coagulation factors are upregulated in microglia both in an *in vivo* setting and an *in vitro* setting.

### 3.2. Blockade of Thrombin Activity Reduces the Expression of Inflammation and Coagulation Markers in the Hippocampus

**Figure 2:**
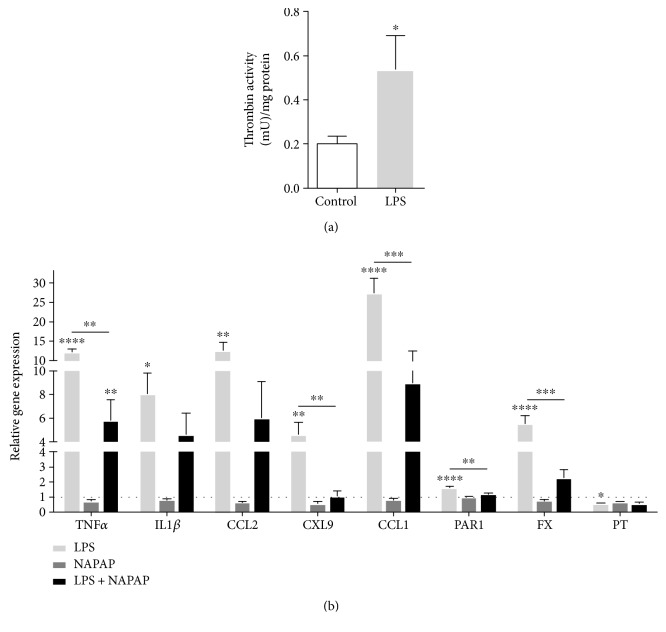
NAPAP treatment modifies hippocampal gene expression following LPS activation. Thrombin activity measured in the hippocampus (mU) and normalized to mg protein (a). *n* = 6, *t-*test. Gene expression analysis of the hippocampus from mice treated with NAPAP and LPS (b). *n* = 6, one-way ANOVA. ^∗^*p* ≤ 0.05, ^∗∗^*p* ≤ 0.01, ^∗∗∗^*p* ≤ 0.001, and ^∗∗∗∗^*p* ≤ 0.0001

The activity of the central coagulation factor thrombin was found to be significantly elevated in the hippocampus following systemic LPS injection (0.5 ± 0.15 mU/mg versus 0.2 ± 0.03 mU/mg in the control, *p* ≤ 0.03, Figure 2(a)). Such result raised the question whether the upregulation of inflammatory cytokines and coagulation factors in the LPS-challenged microglia cells may mirror a situation occurring in the brain. We decided to check the gene expression of those factors in the whole hippocampus. Upon 24 hours from LPS injection, TNF*α*, IL1*β*, CCL2, CXL9, and CCL1 reached levels of 12.2 ± 0.8, 8 ± 1.8, 12.5 ± 2.2, 4.58 ± 1, and 27.3 ± 3.8 (*p* ≤ 0.01), respectively, compared to their respective controls (Figure 2(b)). Factor X and PAR1 gene expression were as well upregulated to 5.5 ± 0.6 and 1.6 ± 0.1, (*p* ≤ 0.0001), respectively, compared to control (Figure 2(b)).

In the periphery, inflammation and coagulation are strictly interconnected [15]; therefore, we decided to evaluate whether blocking thrombin activity may affect the expression of both the inflammatory and coagulation markers. In this experiment, immediately after LPS challenge, mice received NAPAP, an irreversible blocker of thrombin activity. In this setting, NAPAP was able to significantly reduce the expression of both inflammatory and coagulation markers (Figure 2(b)). Indeed, in animals treated with NAPAP and LPS, the expression of TNF*α*, CXL9, and CCL1 dropped to levels of 5.8 ± 1.7, 1 ± 0.3, and 9 ± 3.5 (*p* ≤ 0.005), compared to their respective LPS-injected control (Figure 2(b)). IL1*β* and CCL2 decreased in a nonsignificant manner to 4.6 ± 1.7 and 6 ± 3 compared to their respective LPS-injected control (Figure 2(b)). Factor X and PAR1 gene expression were downregulated to 2.2 ± 0.5 and 1.2 ± 0.05, *p* ≤ 0.003, respectively, compared to LPS-injected control (Figure 2(b)).

Overall, the gene expression profile in the hippocampus (Figure 2(b)) shares a similar pattern to the one obtained in the cells (Figures 1(a)-1(b)), that is, TNF*α*. Nevertheless, a difference was noted with respect to the levels of expression in the different settings. This may possibly be due to the cellular composition of the tested preparations, with the hippocampus containing a wide range of cells (i.e., neurons, etc.) rather than only glia.

## 4. Discussion

In this manuscript, we report that an LPS challenge upregulated the expression of both inflammatory and coagulation factors in microglia and in the hippocampus. Strikingly, inhibiting thrombin activity resulted in a downregulation of inflammatory and coagulation factors in the brain.

LPS has been shown to activate coagulation and inflammation in the periphery [31]. Here, we show that a systemic LPS inflammatory drives the gene expression of inflammation and coagulation factors and their protein synthesis in the brain as well. Microglia seem to be directly involved in this process. It is tempting to speculate about the mechanisms in charge of this phenomenon. A possibility could be that upon LPS exposure, activated macrophages in the periphery may cross the blood-brain barrier (BBB) [31], change their conformation into microglia, and orchestrate the inflammatory response [31, 33]. Alternatively, LPS may break the BBB [34, 35], get into the brain, and activate the resident microglia. More experiments addressing the time scale of microglia activation following a systemic LPS treatment may help in elucidating these mechanisms.

In the periphery, systemic inflammation and coagulation are directly linked [15, 16]. Inflammation initiates clotting while decreasing the activity of natural anticoagulant mechanisms and impairing the fibrinolytic system [15, 22]. Inflammatory cytokines are the major mediators involved in the activation of coagulation [4, 10, 15]. The natural anticoagulants function to dampen the elevation of cytokine levels [15, 36]. Furthermore, components of the natural anticoagulant cascades, like thrombomodulin, minimize endothelial cell dysfunction by rendering the cells less responsive to inflammatory mediators [37], facilitate the neutralization of some inflammatory mediators, and decrease the loss of endothelial barrier function [36, 38, 39]. Hence, downregulation of anticoagulant pathways not only promotes thrombosis but also amplifies the inflammatory process [40, 41]. When the inflammation-coagulation interactions overwhelm the natural defense systems, catastrophic events occur, such as in severe sepsis or in autoimmune diseases [15, 16].

Our data suggest that a crosstalk between inflammation and coagulation may occur in the brain as well. However, the implications for such interactions are far less clear. On the one side, neuroinflammation has profound physiological implications in protecting the brain from internal and/or external injuries [42, 43]. On the other side, amplification of the neuroinflammatory response has been linked to the pathophysiology of stroke and several neurodegenerative diseases [44]. The role of coagulation factors in the brain has only started to be unrevealed. Thrombin in the brain is implicated in synaptic plasticity and learning and memory [45–49], yet upregulation of thrombin in the brain has been linked to seizures [46, 47, 50], maladaptive synaptic plasticity [49, 51], and brain death [52]. How neuroinflammation and brain coagulation interact among themselves is currently unknown. Several data have reported that in neurological diseases, both inflammation and coagulation are upregulated in the brain [7, 27, 53]. In ischemic and hemorrhagic stroke, microglia are recruited to the site of injury where they mediate neuronal death [54] and contribute to brain recovery [44]. In these settings, the upregulated brain concentrations of thrombin have been linked to neuronal damage [26, 29, 30, 55, 56]. In Alzheimer's disease and in vascular dementia, high levels of thrombin and other coagulation factors have been detected in the brain aside with the recruitment of microglia and additional inflammatory components [57–59].

The evidence that the bidirectional relationship between coagulation and inflammation plays a pivotal role in the mechanisms leading to organ failure in patients with severe infection or sepsis has promoted the theory that a pharmacological restoration of the anticoagulant mechanisms may be a logical action in the treatment of septic patients with coagulation abnormalities [60]. Our experiments show that, in the brain, blockade of thrombin activity (i.e., and consequently of the coagulation system) may reduce neuroinflammation and possibly limit the neuronal damage associated to it. If several experimental and initial clinical studies have been started to better address the interaction between inflammation and coagulation in the periphery, similarly, additional studies in the brain are needed. Better investigating the crosstalk between neuroinflammation and coagulation in the brain may result in the development of novel therapeutic strategies for brain disorders.

## Figures and Tables

**Table 1:** 

Gene	Forward	Reverse
HPRT	GATTAGCGATGATGAACCAGGTT	CCTCCCATCTCCTTCATGA CA
PT (prothrombin)	CCGAAAGGGCAACCTAGAGC	GGCCCAGAACACGTCTGTG
FX (factor X)	GTGGCCGGGAATGCAA	AACCCTTCATTGTCTTCGTTAATGA
PAR1	TGAACCCCCGCTC ATTCTTTC	TGAACCCCCGCTC ATTCTTTC
EPCR	ATGTGGCCGTGAATGGAAGCGC	CCATCAGGATGCCCAGGACC
TNF*α*	GACCCTCACACTCAGATCATCTTCT	CCTCCACTTGGTGGTTTGCT
IL1*β*	CTGGTGTGTGACGTTCCCATTA	CCGACAGCACGAGGCTTT
CCL2	CCGGCTGGAGCATCCACGTGT	TGGGGTCAGCACAGACCTCTCTCT
CXL9	TCCTTTTGGGCATCATCTTCC	TTTGTAGTGGATCGTGCCTCG
CCL1	CACAGGGGCGCCTATCGCCAA	CAAGGCAAGCCTCGCGACCAT

## Data Availability

The data used to support the findings of this study are available from the corresponding author upon request.
